# Desulfurization Efficiency Preserved in a Heterometallic MOF: Synthesis and Thermodynamically Controlled Phase Transition

**DOI:** 10.1002/advs.201802056

**Published:** 2019-02-08

**Authors:** Yi Han, Michael A. Sinnwell, Simon J. Teat, Maria L. Sushko, Mark E. Bowden, Quin R. S. Miller, Herbert T. Schaef, Lili Liu, Zimin Nie, Jun Liu, Praveen K. Thallapally

**Affiliations:** ^1^ Key Laboratory of Eco‐Chemical Engineering College of Chemistry and Molecular Engineering Qingdao University of Science and Technology Qingdao 266042 P. R. China; ^2^ Pacific Northwest National Laboratory Richland WA 99352 USA; ^3^ Advanced Light Source Lawrence Berkeley National Laboratory Berkeley CA 94720 USA

**Keywords:** desulfurization, metal‐organic frameworks, phase transitions, preserved adsorption, purification

## Abstract

Efficient removal of heterocyclic organosulfur compounds from fuels can relieve increasingly serious environmental problems (e.g., gas exhaust contaminants triggering the formation of acid rain that can damage fragile ecological systems). Toward this end, novel metal‐organic frameworks (MOFs)‐based sorbent materials are designed and synthesized with distinct hard and soft metal building units, specifically {[Yb_6_Cu_12_(OH)_4_(PyC)_12_(H_2_O)_36_]·(NO_3_)_14_·*x*S}*_n_* (QUST‐81) and {[Yb_4_O(H_2_O)_4_Cu_8_(OH)_8/3_(PyC)_8_(HCOO)_4_]·(NO_3_)_10/3_·*x*S}*_n_* (QUST‐82), where H_2_PyC = 4‐Pyrazolecarboxylic acid. Exploiting the hard/soft duality, it is shown that the more stable QUST‐82 can preserve desulfurization efficiency in the presence of competing nitrogen‐containing contaminate. In addition, thermodynamically controlled single‐crystal‐to‐single‐crystal (SC–SC) phase transition is uncovered from QUST‐81 to QUST‐82, and in turn, mechanistic features are probed via X‐ray diffraction, inductively coupled plasma atomic emission spectroscopy, and ab initio molecular dynamics simulations.

Gasoline and diesel contain significant levels of heterocyclic organosulfur compounds, such as 4,6‐dimethydibenzothiophene (DMDBT), dibenzothiophene (DBT), and benzothiophene (BT). Such contaminants lead to serious environmental and health issues; namely, exhaust gases SO*_x_* eventually contribute to the formation of acid rain and harmful particulate matter. To address such problems, fuel producers are required to reduce sulfur levels to less than 10 ppmw S.^1^, ^2^, ^3^ Current methods rely on catalytic hydrodesulfurization—a high‐cost, high‐temperature/pressure catalytic process that consumes a great deal of hydrogen.^4^, ^5^, ^6^ A promising alternative method of sulfur mitigation is based on the adsorptive removal of organosulfur compounds, a difficult prospect considering the complicated mixture of contaminates in fuels.^7^, ^8^, ^9^, ^10^, ^11^

In this context, metal‐organic frameworks (MOFs), a class of crystalline porous materials^12^, ^13^, ^14^ composed of metal building units connected by organic linkers via strong coordination bonds, have emerged for broad potential applications in adsorption and separation.^15^, ^16^ Particularly, MOF structures offer significant advantages over traditional porous materials, such as mesoporous silica and activated carbon. Potential of MOFs for adsorptive desulfurization has been recognized and carried forward by a number of other scientists and engineers.^17^, ^18^, ^19^ The resulting research showed: i) nitrogen‐containing heterocycle compounds, such as (substituted) indoles or carbazoles, are known to compete for the same adsorption sites as sulfur derivatives^20^, ^21^; ii) MOFs with open metal sites (OMSs) are found to be more efficient for the adsorption of organosulfur or nitrogen compounds, possibly due to sulfur/nitrogen•••metal interactions^22^; and iii) soft organosulfur prefer to interact with softer Lewis acid sites (i.e., Cu^2+^, Zn^2+^, Co^2+^_,_ or Ni^2+^), and the harder bases (nitrogen aromatics) preferentially interact with hard Lewis acid sites (i.e., Fe^3+^, Cr^3+^, or Al^3+^)—in accordance with Pearson's hard–soft acid‐base (HSAB) theory.^23^

To date, only homo‐MOFs have been employed in desulfurization or denitrogenation applications.^19^ MIL‐100 and MIL‐101 (Fe^3+^, Cr^3+^, or Al^3+^), with hard Lewis acid sites for instance, have excellent adsorptive capacities for nitrogen contaminants from simulated fuels; however, these MOFs lack an ability to uptake organosulfur.^23^ In contrast, HKUST‐1 and CPO‐27, or some other materials based on Cu^+^Y zeolite and Mo‐W‐Ni with soft Lewis acid metal sites, could adsorb organosulfur, however decreases desulfurization efficiency due to the competitive adsorption of nitrogen compounds.^23^ Therefore, the rational design and synthesis of specific heterometallic MOFs, in which both distinct hard‐ and soft‐metal building units are periodically segregated among a crystallographic net,^24^, ^25^, ^26^, ^27^, ^28^, ^29^, ^30^, ^31^, ^32^, ^33^, ^34^, ^35^, ^36^, ^37^, ^38^ would be an ideal alternative to preserve desulfurization efficiency in the presence of competing nitrogen compounds.

To this end, herein we report a novel heterometallic MOF, QUST‐81 (where QUST = Qingdao University of Science and Technology), having the formula {[Yb_6_Cu_12_(OH)_4_(PyC)_12_(H_2_O)_36_]·(NO_3_)_14_·*x*S}*_n_* (where S = unassigned free solvent molecules). Surprisingly, QUST‐81 could be transformed into a new MOF, QUST‐82 with formula {[Yb_4_O(H_2_O)_4_Cu_8_(OH)_8/3_(PyC)_8_(HCOO)_4_]·(NO_3_)_10/3_·*x*S}*_n_*, in a single‐crystal‐to‐single‐crystal (SC–SC) manner by heating single crystals of QUST‐81 in the reaction mixture of origin at an increased temperature (120 °C). Importantly, both MOFs possess identical channel sizes and topological nets; however, structural changes can be observed in the metal nodes via single‐crystal X‐ray diffraction (SCXRD). We further probed mechanistic features of the thermodynamically controlled SC–SC phase transition via inductively coupled plasma atomic emission spectroscopy (ICP‐AES) and ab initio molecular dynamics (AIMD) simulations. To our knowledge, we are the first to describe such a phase transition in a heterometallic MOF. In addition, the air‐stable QUST‐82 exhibits great promise for the absorptive removal of prototypical organosulfur compounds, such as DMDBT, DBT, and BT, as well as a nitrogen‐containing contaminate, indole (IND). Furthermore, the desulfurization efficiency could be preserved in the presence of competing nitrogen compounds—the first example for porous materials. We hypothesized the preserved adsorption capacity toward organosulfur to the dual hard/soft Lewis acid nature of QUST‐82.

Solvothermal reactions of 4‐Pyrazolecarboxylic acid (H_2_PyC), Yb(NO_3_)_3_·5H_2_O, and Cu(NO_3_)_2_·2.5H_2_O in a mixture of *N*,*N*‐dimethylformamide (DMF)/*N*,*N*‐dimethylacetamide (DMA)/ *N*‐methyl‐2‐pyrrolidone (NMP) at 100 °C yielded greenish blue single crystals of QUST‐81. SCXRD analysis done at the Advanced Light Source (ALS) revealed that the MOF crystallizes in the cubic space group *Fm*‐3*m* and has a 3D network possessing interconnected nanotubular channels with a pore size of 18 Å. The network is constructed from two distinct and periodic secondary building units (SBUs) (**Figure** **1**a): the classic triangular Cu_3_(OH)(PyC)_3_ SBU (composed of Cu^2+^ and N atoms of the pyrazolate), and a paddle‐wheel Yb_2_(COO_4_)_4_(H_2_O)_8_ SBU (generated from Yb^3+^ and COO^−^ from PyC^2−^). The rare earth–based paddle‐wheel SBU is uncommon in MOFs; however, it was first observed in a photoluminescent europium organic framework.^39^ As a consequence of the assembly process, QUST‐81 possesses two kinds of cages (Figure 1b): Cage I, an octahedral cage of diameter 13 Å composed of four triangular Cu_3_(OH)(PyC)_3_ and six paddle‐wheel Yb_2_(COO_4_)_4_ units. Cage II is 23 Å in diameter residing in the body center of four Cage I, decorated with eight Cu_3_(OH)(PyC)_3_ and twelve Yb_2_(COO_4_)_4_ SBUs. The topology of QUST‐81 is ***pcu***, in which Cage I and paddle‐wheel Yb_2_(COO_4_)_4_ SBU are respectively recognized as 6‐ and 2‐coordinated nodes (Figure 1c; Figure S1, Supporting Information).^40^ The solvent‐accessible volume in QUST‐81 is estimated to be 79.9% after removing all of disordered guest solvent molecules and NO_3_^−^.^41^

**Figure 1:**
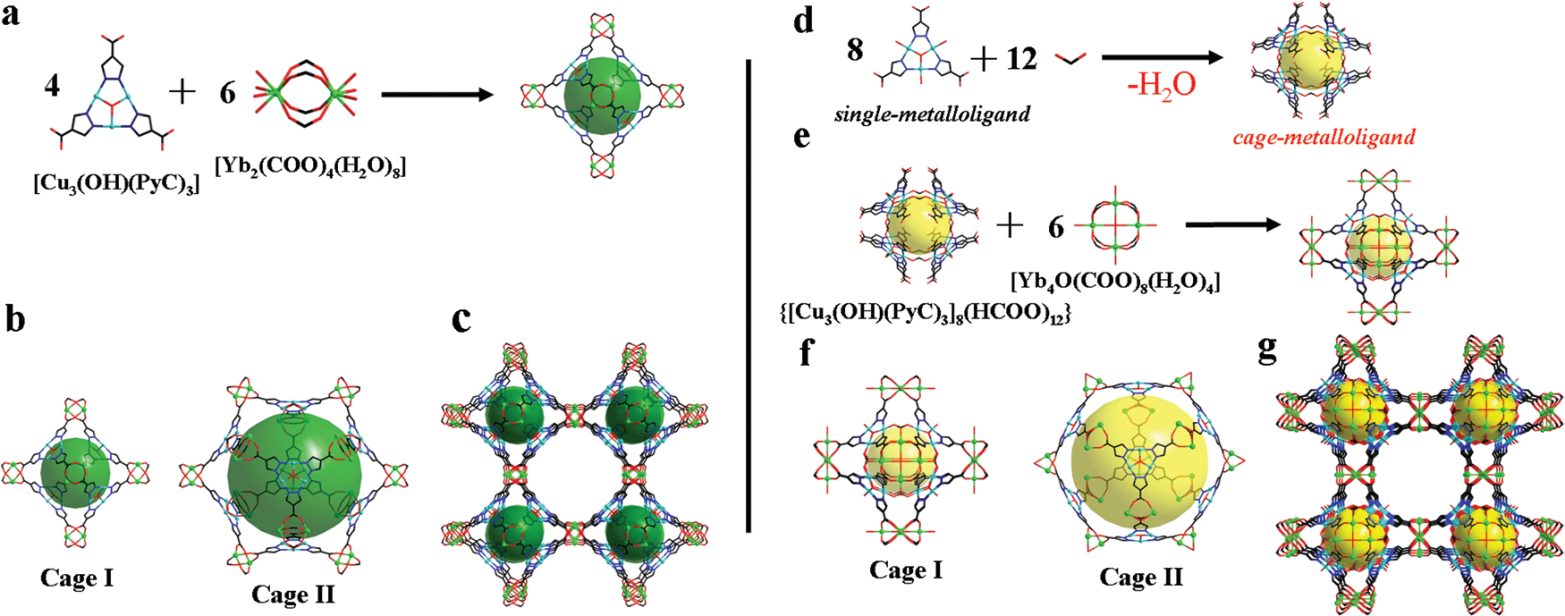
X‐ray crystal structure of QUST‐81, showing a) distinct heterometallic SBUs, b) cages, and c) 3D framework with ***pcu*** topology. Crystal structure of QUST‐82, highlighting structural differences and similarities between QUST‐81 and QUST‐82 in the d) cage‐metalloligand, e) SBUs, f) cages, and g) ***pcu*** topology. Atom colors: Yb = green; Cu = light blue; O = red; C = black; N = dark blue. Parts of coordinated water molecules are omitted for clarity.

The as‐synthesized QUST‐81 gradually lost its crystallinity under solvent‐free conditions, as confirmed by powder X‐ray diffraction (PXRD). However, a new, stable crystalline phase (herein, QUST‐82) formed when the single crystals of QUST‐81 were reheated at an increased temperature of 120 °C in the original reaction solution (Figure S2a, Supporting Information). During this process, we observed neither dissolution nor recrystallization of the single crystals. Scanning electron microscopy (SEM) further confirmed similarities in the size and shape of single crystals of QUST‐81 and QUST‐82 (**Figure** **2**a), indicating a possible SC–SC transformation. Further evidence for the SC–SC process comes from the observation that *single crystals* of QUST‐82—suitable for SCXRD—could only be obtained through single crystals of QUST‐81. *Bulk‐powder* of QUST‐82, however, can be directly synthesized by similar reaction conditions as QUST‐81, a notable difference being a 20 °C increases in the reaction temperature (Figure 2b).

**Figure 2:**
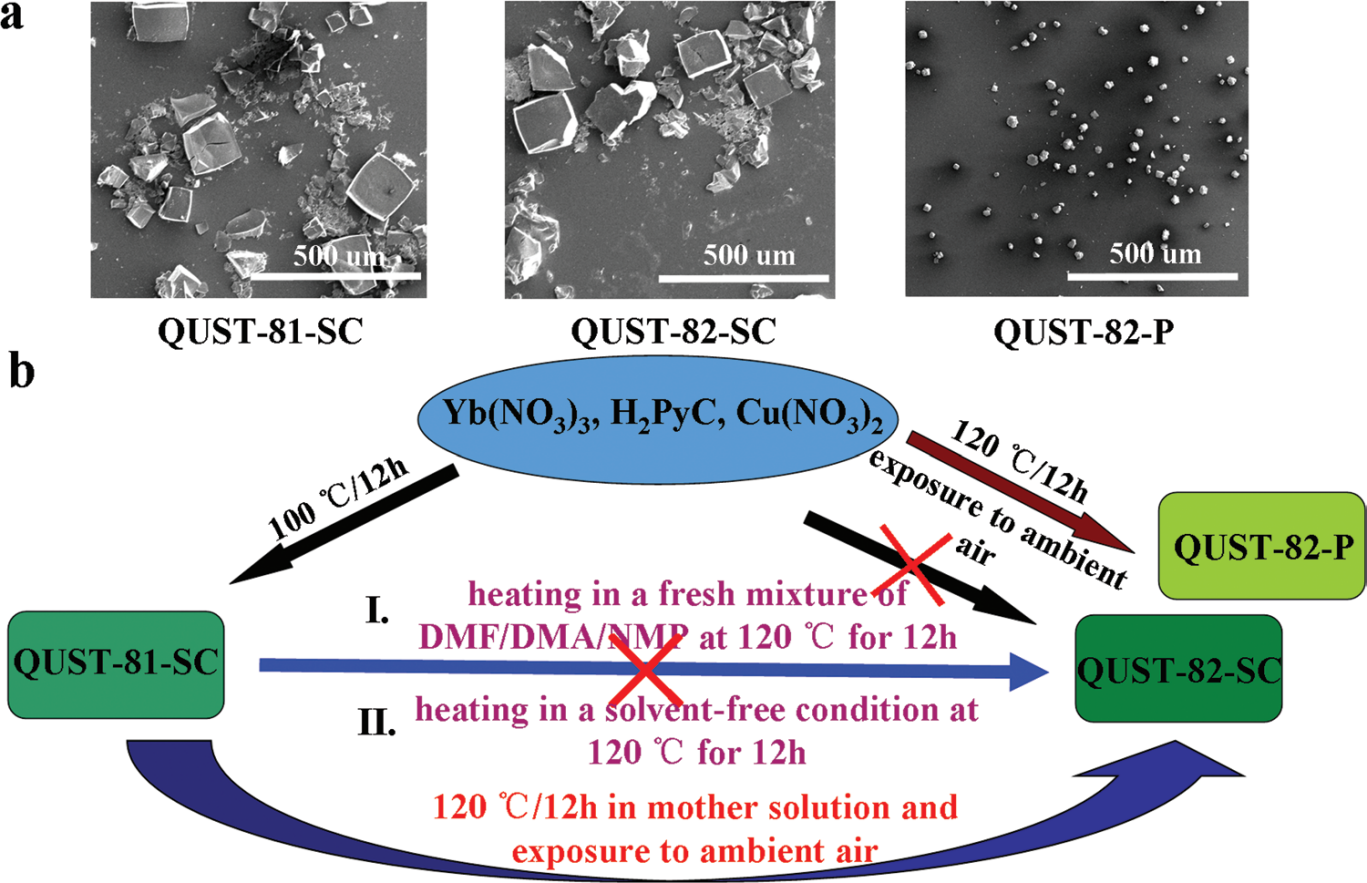
a) SEM images of QUST‐81‐SC, QUST‐82‐SC, and QUST‐82‐P (SC = single crystal and P = powder). b) The process of SC–SC phase transition between QUST‐81 and QUST‐82.

Interestingly, the dark green crystals of QUST‐81 turned colorless during the process before it turned back to green crystals, suggesting that Cu^2+^ in QUST‐81 was first reduced to Cu^+^, and then oxidized back to Cu^2+^ upon exposure to ambient air when QUST‐82‐SC was isolated (Figure S4, Supporting Information).^27^ Notably, we observed colorless intermediates both in the SC–SC transformation and direct synthesis. PXRD indicated that the structural features of the colorless intermediates are similar to those of QUST‐82 (Figure S2a, Supporting Information).

To elucidate mechanistic features of the phase transition, single crystals of QUST‐81 were heated at 120 °C for 12 h in a fresh mixture of DMF/DMA/NMP and also in a solvent‐free condition (Figure 2b). PXRD showed that both resulting products displayed similarities to QUST‐81 (Figure S2b, Supporting Information), suggesting that the excess of Yb^3+^ in the original reaction solution plays a key role in the formation of QUST‐82. Indeed, when we examined the change in Yb^3+^ in the original solution via ICP‐AES, we noticed a decrease in the concentration of Yb^3+^ from 325.2 to 236.8 ppm, possibly suggesting a change in the Yb^3+^‐based SBU of QUST‐81 occurring in the transformation. This was further confirmed by SCXRD.

SCXRD analysis revealed that the SC–SC transformation, in this case, involved the breaking and formation of new coordination bonds, resulting in changes to the coordination environment of the SBUs in QUST‐82. The triangular SBU Cu_3_(OH)(PyC)_3_ remained intact; however, the coordinated water molecules on the Cu ions were substituted by HCOO^−^, generated from the decomposition of DMF,^42^ to form a novel cage‐based metalloligand [Cu_3_(OH)(PyC)_3_]_8_(HCOO)_12_ (Figure 1d). Also, the 8‐coordinated Yb^3+^ in QUST‐81 changed to 6‐coordinated, resulting in a remarkable structural rearrangement from the original paddle‐wheel Yb_2_(COO)_4_(H_2_O)_8_ to a squared Yb_4_O(COO)_8_(H_2_O)_4_ SBU. Despite several reports, by us and others, on SC–SC transformations by external stimuli,^27^, ^43^, ^44^, ^45^, ^46^, ^47^, ^48^, ^49^ this is the first example of a heterometallic MOF in which both distinct metal‐containing building units underwent remarkable transformations that are unambiguously characterized by SCXRD.

AIMD simulations performed at 60 and 120 °C revealed that the possible mechanism for a temperature‐induced phase transition between the paddle‐wheel Yb_2_(COO_4_)_4_(H_2_O)_8_ SBU and the squared Yb_4_O(COO)_8_(H_2_O)_4_ SBU is related to the elongation of the [Yb—O] bonds upon heating. As shown in **Figure** **3**, the average bond length for [Yb—O]_A_ bonds is equal to 2.08 Å at 60 °C. These bonds are shorter than those of type B bonds, [Yb—O]_B_ = 2.15 Å. The resultant higher strength of type A bonds suggests that only limited substitution of these bonds by carboxyls is energetically favorable, making the paddle‐wheel SBU more stable at 60 °C. In contrast, at 120 °C, the relative stability of type A and B bonds is reversed. The bonds in the paddle‐wheel SBU stretch and the average length of type A bonds becomes 2.29 Å, while type B bonds stretch only to 2.21 Å. This reversed stability of Yb—O bonds favors a higher level of O substitution with COO^−^. Furthermore, this substitution along with the formation of a squared Yb_4_O(COO)_8_(H_2_O)_4_ SBU facilitates Yb—O bond contraction and stabilization. The corresponding bond lengths in the squared SBU are equal to 2.16 and 2.09 Å for [Yb—O]_A_ and [Yb—O]_B_, respectively.

**Figure 3:**
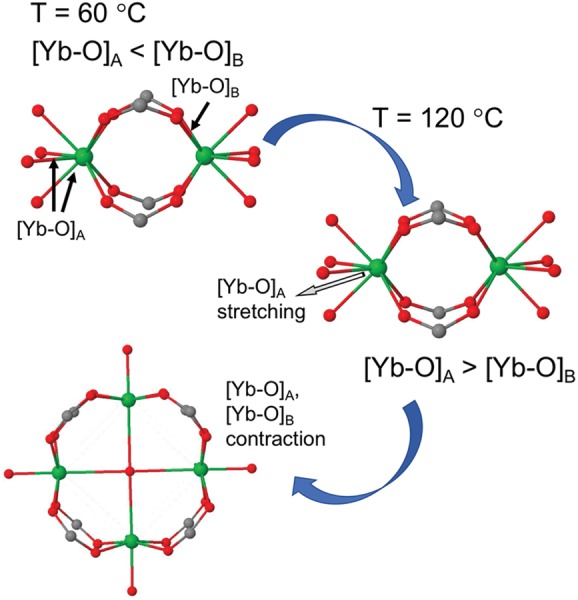
Simulated mechanism of the temperature‐induced phase transition between the paddle‐wheel Yb_2_(COO_4_)_4_(H_2_O)_8_ and squared Yb_4_O(COO)_8_(H_2_O)_4_ SBUs. Yb—O bond length analysis indicates that the paddle‐wheel SBU has higher stability at 60 °C and the squared one has higher stability at 120 °C.

In addition to the changes to the SBUs, XRD analysis revealed that QUST‐82, which crystallizes in the cubic space group *Pm*‐3*m*, also contains two distinct cages: octahedral cage I with 15 Å in diameter defined by six Yb_4_O(COO)_8_(H_2_O)_4_ and one [Cu_3_(OH)(PyC)_3_]_8_(HCOO)_12_. Cage II is similar in terms of shape and chemical composition to that in QUST‐81, but slightly larger in size (25 vs 23 Å) (Figure 1f). Compared with QUST‐81 (79.9%), the solvent‐accessible volume in QUST‐82 decreased to 71.9%,^41^ possibly due to the structural rearrangement. In spite of these, QUST‐82 also possesses ***pcu*** topology with an approximate pore size of 18 Å (Figure 1g; Figure S1, Supporting Information).^40^ Oriented toward the channel centers or located on the surface of the pores are both the hard Yb^3+^ and soft Cu^2+^ metal sites. The location of these sites suggests an increased probability that a foreign guest can interact with either a hard or a soft metal site for possible adsorption and separation applications.

To assess the permanent porosity of QUST‐82, N_2_ adsorption experiment at 77 K was performed after solvent‐exchange with acetone. The N_2_ adsorption isotherms of QUST‐82 showed characteristic type‐I behavior with Brunauer–Emmett–Teller (BET) and Langmuir surface area of 2148 and 2733 m^2^ g^−1^, as well as a pore volume of 0.964 cm^3^ g^−1^, respectively (Figure S5 and Table S3, Supporting Information). PXRD indicated that QUST‐82's structure remains intact after N_2_ adsorption. In addition, QUST‐82 can be stable in common organic solvents, however lost its crystallinity when exposed to water (Figure S2, Supporting Information). Nevertheless, the permanent porosity, stability, pore size, and accessibility of both hard/soft metal sites inspired us to investigate QUST‐82 for desulfurization and denitrogenation applications.

To do so, we first measured single‐component adsorption isotherms for S‐containing DMDBT, DBT, and BT, as well as for N‐containing IND in isooctane by UV/vis spectra at a variety of concentrations (Table S2, Supporting Information). For example, QUST‐82 exhibits excellent capacities at high concentrations (1500 ppmw S for DBT and BT, 600 ppmw S for DMDBT [due to low solubility], or 46 mm for IND; Figures S7–S10 and Table S2, Supporting Information) with DBT capacity of 262 (g DBT kg^−1^ MOF); BT capacity of 116 (g BT kg^−1^ MOF); DMDBT capacity of 115 (g DMDBT kg^−1^ MOF); and IND capacity of 219.4 (g IND kg^−1^ MOF), respectively. For comparison with those previously reported benchmark MOFs, QUST‐82 shows higher adsorption capacity for DBT (g DBT kg^−1^ MOF, 1500 ppmw S) than MOF‐177 (≈92),^17^ CPO‐27 (Ni) (158), MOF‐5 (≈161),^17^ Cu_3_(NAPANA) (187),^50^ and MOF‐505 (225),^17^ similar to that of HKUST‐1 (259),^17^ and only lower than UMCM‐150 (478).^17^ For BT (g BT kg^−1^ MOF, 1500 ppmw S), QUST‐82 displays higher adsorption capacity than MOF‐177 (≈37),^17^ MOF‐5 (≈51),^17^ and HKUST‐1 (105),^17^ similar to Cu_3_(NAPANA) (117),^50^ and lower than UMCM‐150 (169)^17^ and MOF‐505 (215).^17^ For DMDBT (g DMDBT kg^−1^ MOF, 600 ppmw S), QUST‐82 possesses higher adsorption capacity than HKUST‐1 (106),^17^ and lower than UMCM‐150 (272)^17^ and MOF‐505 (179).^17^ For IND (g IND kg^−1^ MOF, 46 mm), we found that QUST‐82 exhibits higher adsorption capacity (219.4) than HKUST‐1 (206.4) and CPO‐27 (Ni) (105.5) observed in this work.

Typically, the differences in the adsorption capacities listed here are attributed to the correlations between OMS, surface area, and pore volume among the related MOFs (Table S3, Supporting Information). However, the saturation has not been reached for all the three isotherms for DBT, BT, and IND, suggesting that QUST‐82 has the potential for even greater adsorption (Figure S11, Supporting Information). Clearly, the adsorption amount of DBT at equilibrium at any investigated concentrations is higher than those recorded for BT (Figure S10, Supporting Information). We attributed the lower BT adsorption to a combination of interactions between OMS (coordination) and electron‐rich organosulfur compounds, and π–π interactions between conjugated organosulfur compounds and aromatic ligand used in this work.^22^, ^23^, ^51^ In addition, the N_2_ adsorption isotherms at 77 K of DBT@QUST‐82, BT@QUST‐82, and IND@QUST‐82 showed decreases in the BET surface area and pore volume as a result of DBT, BT, or IND uptake (Figure S5 and Table S4, Supporting Information), suggesting that QUST‐82 pores are accommodating the DBT, BT, or IND guests.

Next, we investigated the reversibility of QUST‐82; our results indicate full regeneration is feasible via the exchange of organosulfur and nitrogen compounds with a polar solvent such as ethanol, as confirmed by UV/vis and N_2_ adsorption studies. The UV/vis spectra show that DBT, BT, and IND molecules trapped in QUST‐82 can be completely released within 48 h (Figure S13, Supporting Information). The BET surface‐area and pore‐volume values of as‐synthesized and regenerated QUST‐82 are very similar (Figure S5 and Table S4, Supporting Information). PXRD demonstrated that regenerated QUST‐82 maintains its original structural characteristics after one run of the regeneration measurement (Figure S2c, Supporting Information). After being used for adsorptive desulfurization (900 ppmw S for DBT or BT) or denitrogenation (28 mm for IND) for a second run, the regenerated QUST‐82 retained its adsorption capabilities (Table S2, Supporting Information). Thus, we concluded that QUST‐82 has potential as a cost‐effective adsorbent for the removal of organosulfur and nitrogen compounds.

Motivated by the coexistence of hard and soft Lewis acid sites in QUST‐82, its excellent stability, and high single‐component capacity for desulfurization, we further investigated the process of using QUST‐82 to remove organosulfur contaminants (DBT) in the presence of competing nitrogen compounds (IND) at equivalent concentrations for the first time. In this case, HKUST‐1 and CPO‐27 (Ni) were selected, both of which can adsorb organosulfur and also nitrogen compounds, for comparison (**Figure** **4**). It was found that, QUST‐82, HKUST‐1, and CPO‐27 (Ni) exhibit DBT uptake capacities of 241, 196, and 80 (g DBT kg^−1^ MOF, DBT/IND at 1500 ppmw S 46 mm ^−1^) and 69.5, 34.8, and 12.3 (g DBT kg^−1^ MOF, DBT/IND at 300 ppmw S 9 mm ^−1^), respectively (note: DBT at 1500 ppmw S = IND at 49 mm; DBT at 300 ppmw S = IND at 9 mm) (Table S5, Supporting Information). As clearly shown in **Figure** **5**, sharp decreases in DBT capacity are observed in HKUST‐1, particularly at the lower concentration; both sharper decreases in DBT capacity are observed in CPO‐27 (Ni) when IND is present. Strikingly, the DBT adsorption capacity of QUST‐82, at both high and low concentrations, changes little in the presence of IND. Compared with homometallic‐MOFs (HKUST‐1 and CPO‐27 (Ni)), the presence of a hard Lewis acid site (Yb^3+^) in QUST‐82 can greatly relieve the competitive adsorption of nitrogen compounds on Cu^2+^ sites due to the preferential interactions between Yb^3+^ and IND, and further using the Cu^2+^ sites to interact with DBT to realize the desulfurization efficiency to be preserved. In addition, a breakthrough experiment on QUST‐82, however, showed a lack of separation ability between DBT and IND, probably indicating that both compounds are adsorbed at a similar rate (Figure S14, Supporting Information). Collectively, the results indicate that the dual hard/soft Lewis acid nature in QUST‐82 contributed to the desulfurization efficiency being preserved in the presence of competing nitrogen contaminants (IND), regardless of relative concentrations.

**Figure 4:**
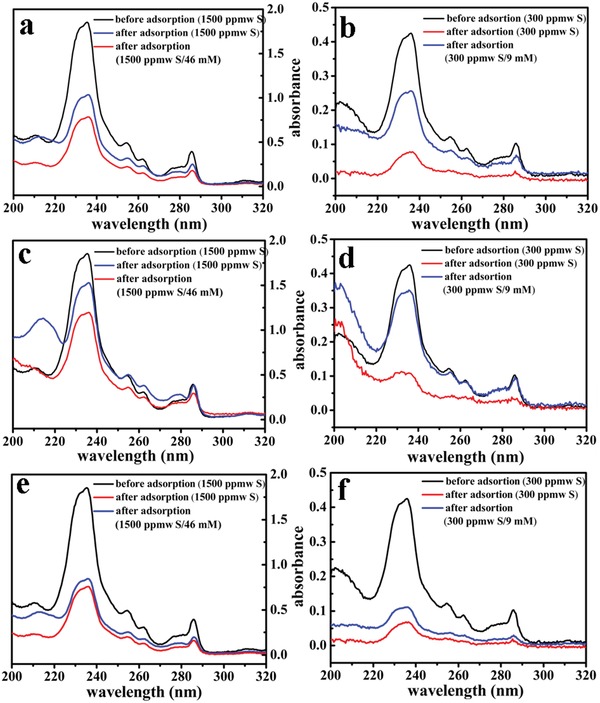
UV/vis spectra of isooctane solution to check the adsorption capacity for DBT of HKUST‐1 a,b), CPO‐27 (Ni) c,d) and QUST‐82 e,f) in pure or mixed systems (DBT/IND).

**Figure 5:**
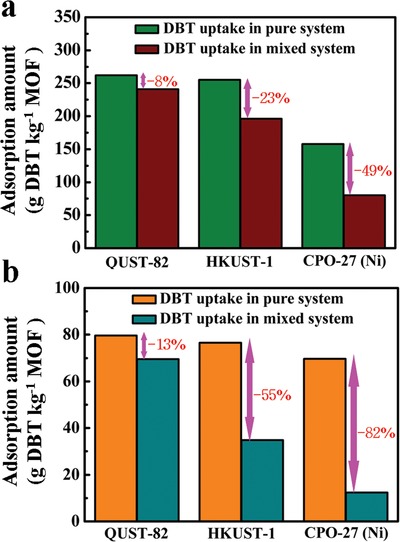
Columnar illustration showing the efficiency for removal of DBT using QUST‐82, HKUST‐1, and CPO‐27 (Ni) in mixed systems with a) DBT/IND = 1500 ppmw S 46 mm ^−1^ or b) DBT/IND = 300 ppmw S 9 mm ^−1^ (note: in pure systems with a) DBT = 1500 ppmw S or b) DBT = 300 ppmw S).

Furthermore, we examined the adsorption capacity of QUST‐82 for IND from the mixture of DBT and IND (1500 ppmw S 46 mm ^−1^ ) (Figure S15, Supporting Information). QUST‐82 could only adsorb 113.5 g IND kg^−1^ MOF, which is 51.7% of the amount of IND adsorbed from pure IND at 46 mm. This dramatic decrease suggests that both Yb and Cu ions can interact with pure IND, whereas in the presence of DBT, the reduction of the accessibility between IND and Cu^2+^ occurs due to the stronger coordination between Cu^2+^ and S in DBT. We also attempted to determine the adsorption capacity of BT from the mixture of BT/IND, but this failed because the characteristic absorption bands of BT and IND merged into one broad band, making them indistinguishable.

In summary, the work described herein demonstrates the thermodynamically controlled, heterometallic MOF QUST‐82 obtained via SC–SC transformation. Surprisingly, even though the bond breaking and formation of new coordination bonds induced structural rearrangements occurring in the process, both MOFs were identical in terms of pore size and topology. QUST‐82 is an excellent adsorbent suitable for the removal of both organosulfur and nitrogen compounds in isooctane. In addition, compared with homometallic‐MOFs HKUST‐1 and CPO‐27 (Ni), QUST‐82 maintained relatively high adsorption capacity for DBT from a mixture of DBT and IND. These results demonstrate the potential for new porous materials predicted to preserve desulfurization efficiency when competing nitrogen contaminants are present. The discoveries in this work represent the beginning of possibly extensive exploration of a unique synthesis approach and potential applications of stable heterometallic MOFs. Continued research into the practical uses of functional heterometallic MOFs is underway.

## Experimental Section

*Synthesis and Activation of MOFs*: QUST‐81: A mixture of Yb(NO_3_)_3_·5H_2_O (196 mg, 0.44 mmol), Cu(NO_3_)_2_·2.5H_2_O (7.7 mg, 0.03 mmol), and H_2_PyC (5 mg, 0.04 mmol) dissolved in DMF (1.5 mL), DMA (1.5 mL), and NMP (1 mL) in a 5 mL vial was heated at 100 °C for 12 h. Greenish blue crystals of QUST‐81 in 86% yield (based on H_2_PyC) were harvested. The resultant framework formula of {[Yb_6_Cu_12_(OH)_4_(PyC)_12_(H_2_O)_36_]·(NO_3_)_14_·*x*S}*_n_* was defined from charge balance.

*QUST‐82*: Heating the single crystals of QUST‐81 in mother solution at 120 °C for 12 h can produce colorless crystals. After loosening the vial cap, the color of the crystals turned dark green to generate QUST‐82. The microcrystalline powder of QUST‐82, however, could be obtained by direct heating of the mixture prepared for QUST‐81 at 120 °C for 12 h (yield: 53%, based on H_2_PyC). As‐synthesized powder sample of QUST‐82 was thoroughly washed by fresh DMF and immersed in DMF (4 mL) for 3 days, during which DMF was decanted and freshly replenished three times. The resulting sample was then soaked in acetone for 5 days, and the acetone was replaced three times per day. The solid was then dried at 85 °C under vacuum for 24 h to yield activated sample. IR (KBr): 3434 (w), 2943 (w), 2326 (w), 1651 (m), 1600 (m), 1550 (s), 1455 (s), 1382 (w), 1295 (s), 1172 (w), 1190 (w), 1063 (m), 1012 (m), 795 (s), 671 (m), 620 (w) cm^−1^; EA (%) of activated sample: Calcd for Yb_4_Cu_8_C_36_H_68/3_N_58/3_O_113/3_ = [Yb_4_OCu_8_(OH)_8/3_(PyC)_8_(HCOO)_4_]·(NO_3_)_10/3_: C, 17.10; H, 0.90; N, 10.7; Found: C, 16.91; H, 1.32; N, 9.86. The resultant framework formula of {[Yb_4_O(H_2_O)_4_Cu_8_(OH)_8/3_(PyC)_8_(HCOO)_4_]·(NO_3_)_10/3_·*x*S}*_n_* was defined from elemental analysis (EA) and charge balance.

*Analytical Methods*: Before the adsorption experiments, the standard curves (A/C) of DBT, BT, and IND in isooctane toward the concentrations of 1500, 1200, 900, 600, and 300 ppmw S for DBT or BT, 600, 500, 400, 300, 200 ppmw S for DMDBT, and 46, 37, 28, 18, and 9 mm for IND were recorded. In a 2 mL glass vial, 0.1 mL solution was added to 1 mL isooctane, and 10 µL (for DBT) or 20 µL (for BT and IND) of the resulting solution was transferred into 1 mL isooctane for direct measurement by UV spectroscopy.

The three function equations are as follows:

A=0.1108+0.00272C for DMDBT

A=0.0374+0.00122C for DBT

A=0.0339+0.00129C for BT

A=−0.10132+0.04502C for IND

where A and C are, respectively, the absorbance and concentration in the resulting solution, which were obtained by linear fit of the data.

A liquid‐phase adsorption experiment from pure systems was carried out at room temperature in 5 mL glass vials filled with activated adsorbent (20 mg) and a solution of isooctane (1 mL) including DMDBT, DBT, BT, or IND at different concentrations. Then, the vials were agitated on a shaker for 5 h. 0.1 mL of the final isooctane solution, after removing the solid adsorbents, was extracted and 1 mL isooctane was added sequentially. 10 µL (for DBT) or 20 µL (for BT and IND) solution was transferred into 1 mL isooctane for direct monitoring by UV spectroscopy (For DMDBT, 0.1 mL of the final isooctane solution, after removing the solid adsorbents, was extracted and 1 mL isooctane was added sequentially. Then, 0.1 mL of the resulting isooctane solution was extracted and 1 mL isooctane was added. 400 µL solution was transferred into 1 mL isooctane for direct monitoring by UV spectroscopy.) Equilibration times were determined for the characteristic UV absorption band of each compound until no further changes. The resulting adsorption amount for each compound at different concentrations was calculated by the above function equations.

For adsorption experiment from mixture systems, 20 mg activated adsorbent was added into 5 mL glass vials filled with a solution of isooctane (1 mL) including DBT/IND (1500 ppmw S 46 mm ^−1^ or 300 ppmw S 9 mm ^−1^). The following procedures can be referred to those for pure systems.

*Regeneration Experiments*: The samples of DBT@QUST‐82, BT@QUST‐82, or IND@ QUST‐82 were soaked in ethanol (4 mL), and replaced with fresh ethanol (4 mL) eight times during 48 h. The regeneration procedures were monitored by UV for each washed ethanol.

[CCDC 1835704 (QUST‐81) and 1827059 (QUST‐82) contain the supplementary crystallographic data for this article. These data can be obtained free of charge from The Cambridge Crystallographic Data Centre via www.ccdc.cam.ac.uk/data_request/cif.]

## Conflict of Interest

The authors declare no conflict of interest.

## Supporting information

Supplementary
