# Supplementary material for: Desulfurization Efficiency Preserved in a Heterometallic MOF: Synthesis and Thermodynamically Controlled Phase Transition
Source: Adv Sci (Weinh). 2019 Feb 8;6(7):1802056. doi: 10.1002/advs.201802056 (PMC6446612; doi:10.1002/advs.201802056)
Supplement: Supplementary file 1 — Supplementary [file ADVS-6-1802056-s001.pdf]

Copyright WILEY-VCH Verlag GmbH & Co. KGaA, 69469 Weinheim, Germany, 2019.

ADVANCED SCIENCE
Open Access

Supporting Information

for *Adv. Sci.,* DOI: 10.1002/advs.201802056

Desulfurization Efficiency Preserved in a Heterometallic MOF: Synthesis and Thermodynamically Controlled Phase Transition

Yi Han, Michael A. Sinnwell, Simon J. Teat, Maria L. Sushko, Mark E. Bowden, Quin R. S. Miller, Herbert T. Schaef, Lili Liu, Zimin Nie, Jun Liu, and Praveen K. Thallapally**

Copyright WILEY-VCH Verlag GmbH & Co. KGaA, 69469 Weinheim, Germany, 2019.

Supporting Information

Desulfurization Efficiency Preserved in a Heterometallic MOF: Synthesis and Thermodynamically-Controlled Phase Transition

Yi Han,* Michael A. Sinnwell, Simon J. Teat, Maria Sushko, Mark E. Bowden, Quin R. S. Miller, Herbert T. Schaef, Lili Liu, Zimin Nie, Jun Liu and Praveen K. Thallapally*

Contents

S1. Materials and general methods..3

S2. Single-crystal and powder X-ray crystallography..4

S3. Synthesis and activation of MOFs..6

S4. Supporting figures..8

S5. Supporting tables and references..16

I. Materials and general methods

$Yb(NO_3)_3 \cdot 5H_2O$ (99.9%, Sigma-Aldrich), $Cu(NO_3)_2 \cdot 2.5H_2O$ (98%, Sigma-Aldrich), $Ni(CH_3COO)_2 \cdot 4H_2O$ (98%, Sigma-Aldrich), 4-Pyrazolecarboxylic acid (H_2PyC) (98%, Combi-Blocks), Benzene-1,3,5-tricarboxylic acid (H_3BTC) (95%, Sigma-Aldrich), 2,5-dihydroxyterephthalic acid (H_2DHTA) (>98%, TCI), Benzothiophene (BT) (99%, Sigma-Aldrich), dibenzothiophene (DBT) (98%, Sigma-Aldrich), Indole (IND) (99%, Sigma-Aldrich), N,N-Dimethylformamide (DMF) (99.9%, Fisher), N,N-Dimethylacetamide (DMA) (≥99.9%, Sigma-Aldrich), N-Methyl pyrrolidone (NMP) (99%, Sigma-Aldrich), tetrahydrofuran (THF) (99.9%, Sigma-Aldrich), dichloromethane (99.8%, Sigma-Aldrich), ethanol (95%, Fisher), and isooctane (HPLC grade, Fisher) were used as received.

IR spectra were recorded from KBr pellets in the range 4000-600 cm^{-1} on a Shimadzu IR435 spectrometer. A FLASH EA 1112 analyzer was performed for the elemental analysis. Powder X-ray diffraction (PXRD) was carried out with an X-ray diffractometer of Rigaku, MiniFlexII using Cu−Kα radiation (λ = 1.5418 Å) or Bruker D8 Discover (Bruker AXS Inc., Madison, WI), equipped with a rotating Cu anode (λ = 1.5418 Å). Thermogravimetric analyses (TGA) were carried out on a NETZSCH TG 209 F1 analyzer heated from room temperature to 800 °C at a ramp rate of 5 K min^{-1} under nitrogen atmosphere. The UV/Vis

absorption spectra were examined on Ultrospec 2100 pro spectrophotometer in the wavelength range of 200-320 nm. Scanning electron microscopy (SEM) was acquired on FEI Helios. The N_2 adsorption measurements were performed on Quantachrome automated gas adsorption analyzer.

II. Single-crystal and powder X-ray Crystallography

Single-crystal X-ray Crystallography

Single crystal of QUST-81 was selected and mounted on MiTeGen® loops in Paratone oil. Cooling the sample to below room temperature resulted in a loss of resolution in the diffraction data. So a drop of mother liquor was placed in a MiTeGen® MicroRT® tube which was then placed over the crystal and loop and secured to the base by the means of grease. This was mounted on a Bruker D8 diffractometer equipped with a PHOTONII CPAD detector at 298 K, on Beamline 12.2.1 of the Advanced Light Source at LBNL. A sphere of data were collected using Bruker APEX3 software in shutterless mode with ω rotations at fixed φ values at λ = 0.7749 Å, from a Silicon [111] double crystal monochromator. The intensity data were integrated and correction applied with SAINT v8.38a, absorption and other correction were made using SADABS 2016/2. Dispersion corrections appropriate for this wavelength were calculated using the Brennan method in XDISP with in WinGX. The structures were solved with a dual space method with SHELXT 2014/5 and refined using SHELXL 2014/7.

The data were cut at 1.16 angstroms as R(int) jump from 30% to 42% in the next shell. Experience has shown that this addition data would add more noise than useful data. All non-hydrogen atoms were refined anisotropically, except the partially occupied water molecules. Hydrogen atoms were found placed geometrical on the carbon atom and refined as a riding model. Hydrogen atoms could neither be found nor placed on the water molecules and were therefore omitted from the refinement but not the chemical formula. Displacement parameter restraints were used globally (RIGU) and same distance restraints were used for Yb1 to O1w and O2w, also for Cu1 to O3w O3w'. Water molecules were used to complete the metal coordination shell as not meaningful electron density could be found in the difference map to such any other option. The same is true of the waters in the void space. CCDC-1835704 (QUST-81) contains the supplementary crystallographic data for this paper.

Data collection for QUST-82 was carried on an Agilent Technologies SuperNova Single Crystal Diffractometer using Cu Kα radiation (λ = 1.54178 Å) at 120 K. The structure was solved using SHELXS-97 and was refined with SHELXL-97. The hydrogen atoms were

included in the structure-factor calculations at idealized positions by using a riding model and were refined isotropically. The contributions of guests were removed by using SQUEEZE as implemented in PLATON. CCDC-1827059 (QUST-82) contains the supplementary crystallographic data for this paper.

powder X-ray Crystallography

In order to analyze the air-sensitive colorless intermediate crystals with PXRD, the fresh solution of starting materials for the synthesis of QUST-81 was placed in a 3.0 mm diameter thin walled borosilicate capillary tube for XRD applications (Charles Supper Company). After the capillary was sealed with room temperature vulcanization epoxy, it was placed in a lab oven at 100 °C to generate QUST-81 (Figure S4) and subsequently heated to 120 °C to generate the intermediate phase with colorless crystals (Figure S4). PXRD analysis of the air-sensitive colorless crystals that settled to the bottom of the sealed capillary was conducted with a Bruker D8 Discover (Bruker AXS Inc., Madison, WI), equipped with a rotating Cu anode (Kα λ = 1.5418 Å), capable of producing an intensely focused 0.5 mm beam. The capillary was mounted on the custom-built programmable XYZ stage and positioned using a laser-video alignment system. A Vantec 500 area detector system positioned at 15.4 °2θ with a measured sample-detector distance of 15 cm was used to capture diffraction images. A lead beam stop was utilized to prevent the beam from saturating the detector and obscuring low angle peaks. Collection of individual XRD tracings required 200 s with power settings of 50 kV and 25 mA. Initially, images were processed with Bruker-AXS GADDS software before importing into MDI JADE XRD software to obtain peak positions and intensities.

III. Synthesis and activation of MOFs

Synthesis of QUST-81

A mixture of $Yb(NO_3)_3{\cdot}5H_2O$ (196 mg, 0.44 mmol), $Cu(NO_3)_2{\cdot}2.5H_2O$ (7.7 mg, 0.03 mmol), and H_2PyC (5 mg, 0.04 mmol) dissolved in DMF (1.5 mL), DMA (1.5 mL) and NMP (1 mL) in a 5 mL vial was heated at 100 °C for 12 h. Greenish blue crystals of QUST-81 in 86% yield (based on H_2PyC) were harvested. The resultant framework formula of $\{[Yb_6Cu_{12}(OH)_4(PyC)_{12}(H_2O)_{36}]{\cdot}(NO_3)_{14}{\cdot}xS\}_n$ was defined from charge balance.

Synthesis of QUST-82

Heating the single crystals of QUST-81 in mother solution at 120 °C for 12 h can produce colorless crystals. After loosening the vial cap, the color of the crystals turned dark green to generate QUST-82. The microcrystalline powder of QUST-82, however, could be obtained by

direct heating the mixture prepared for QUST-81 at 120 °C for 12 h (Yield: 53%, based on H_2PyC). As-synthesized powder sample of QUST-82 was thoroughly washed by fresh DMF and immersed in DMF (4 mL) for 3 days, during which DMF was decanted and freshly replenished three times. The resulting sample was then soaking in acetone for 5 days, and the acetone was replaced three times per day. The solid was then dried at 85 °C under vacuum for 24 h to yield activated sample. IR (KBr): 3434 (w), 2943 (w), 2326 (w), 1651 (m), 1600 (m), 1550 (s), 1455 (s), 1382 (w), 1295 (s), 1172 (w), 1190 (w), 1063 (m), 1012 (m), 795 (s), 671 (m), 620 (w) cm^{-1}; EA (%) of activated sample: Calcd for $Yb_4Cu_8C_{36}H_{68/3}N_{58/3}O_{113/3}$ = $[Yb_4OCu_8(OH)_{8/3}(PyC)_8(HCOO)_4]\cdot(NO_3)_{10/3}$: C, 17.10; H, 0.90; N, 10.7; Found: C, 16.91; H, 1.32; N, 9.86. The resultant framework formula of $\{[Yb_4O(H_2O)_4Cu_8(OH)_{8/3}(PyC)_8(HCOO)_4]\cdot(NO_3)_{10/3}\cdot xS\}_n$ was defined from and elemental analysis (EA) and charge balance. QUST-82 is stable in common organic solvents, such as acetone, MeOH, THF and DMF, however, is unstable in water (Fig. S2c).

Synthesis of HKUST-1

H_3BTC (0.25 g, 1.2 mmol) and $Cu(NO_3)_2\cdot 2.5H_2O$ (0.5 g, 2.2 mmol) were stirred for 15 min in 12.5 mL of solvent consisting of equal parts DMF, ethanol and deionized water in a 20 mL glass jar. The jar was tightly capped and placed in an 85 °C oven for 20 h to yield small octahedral crystals. After decanting the hot mother liquor and rinsing with DMF, the product was immersed in dichloromethane for 3 days, during which the activation solvent was decanted and freshly replenished three times. The solvent was removed under vacuum at 170 °C, yielding the porous material. (J. L. C. Roswell, O. M. Yaghi, J. Am. Chem. Soc. **2006**, 128, 1304.)

Synthesis of CPO-27(Ni)

CPO-27(Ni) was synthesized according to literature by mixing a solution of 0.373 g $Ni(CH_3COO)_2\cdot 4H_2O$ in 10 ml of H_2O with a solution of 0.149 of 2,5-dihydroxyterephthalic acid (H_2DHTA) in 10 g of THF. The mixture is then transferred into a Teflon-lined steel autoclave and placed in an oven at 110 °C for three days. After cooling, the mixture was washed with water and the obtained CPO-27(Ni) was activated by heating it overnight at 100 °C under vacuum. (P. Dietzel, B. Panella, M. Hirscher, R. Blom, H. Fjellvag, Chem. Comm., **2006**, 959).

IV. Supporting figures

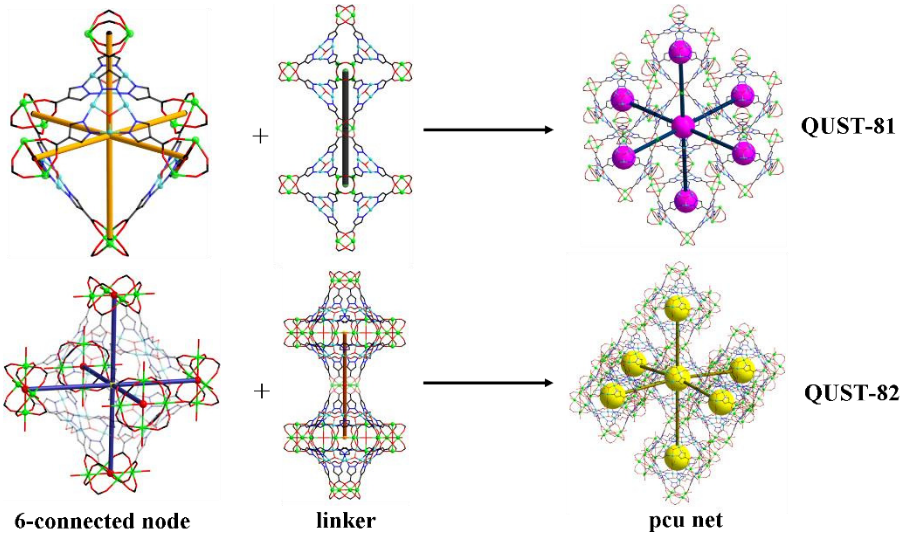

Figure S1. Topological analysis for QUST-81 and QUST-82 calculated by **SYSTRE** (O. Delgado-Friedrichs, Systre version 1.1.2 beta, **2007**.).

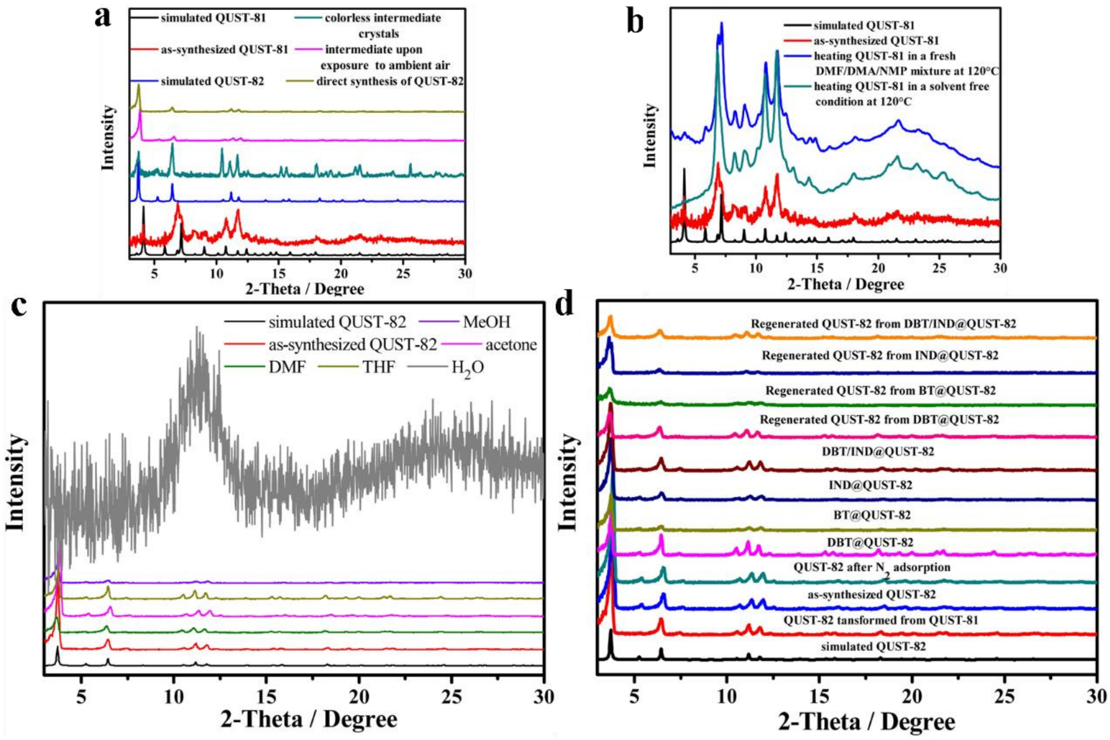

Figure S2. Comparison of the simulated and experimental PXRD patterns of as-synthesized QUST-81, QUST-82 as well as QUST-81 subjected to a phase transition at 120 °C (a); PXRD patterns of QUST-81 (b), QUST-82 (c) and (d) after various treatments .

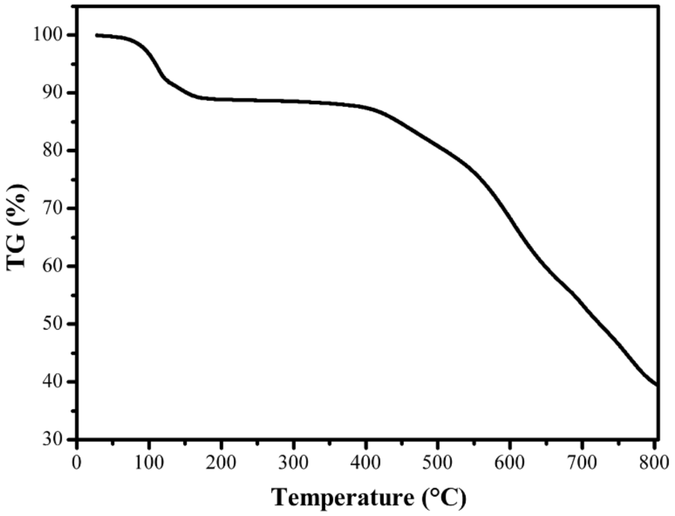

Figure S3. TGA curve of as-synthesized QUST-82.

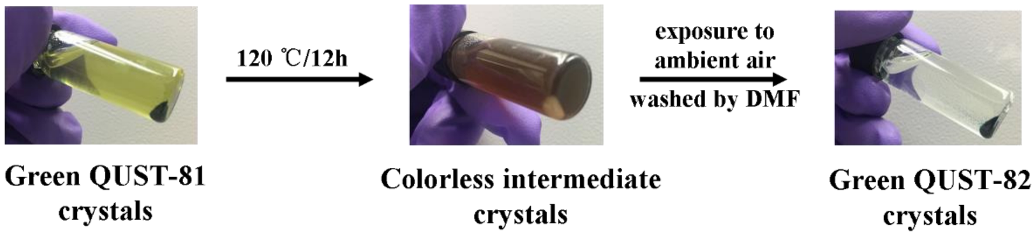

Figure S4. The color changes of the crystals and filtrate during the phase transition.

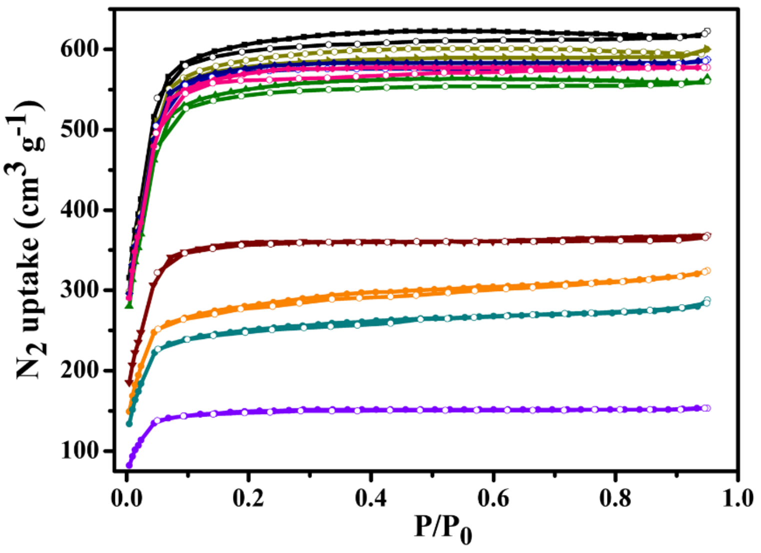

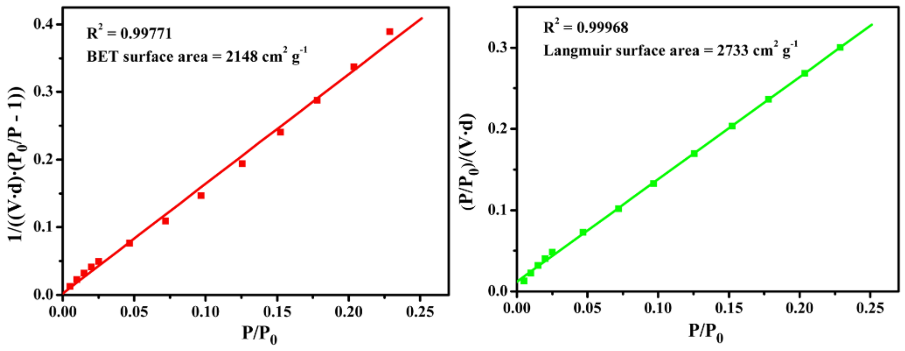

Figure S5. Isotherms for the adsorption of N_2 at 77 K in QUST-82 (balck), DBT@QUST-82 (900 ppmw) (dark cyan), BT@QUST-82 (900 ppmw) (wine), IND@QUST-82 (28 mM) (orange). DBT/IND@QUST-82 (1500 ppmw/46 mM) (violet), regenerated QUST-82 from DBT@QUST-82 (oliver), regenerated QUST-82 from BT@QUST-82 (dark yellow), regenerated QUST-82 from IND@QUST-82 (blue) and regenerated QUST-82 from DBT/IND@QUST-82 (magenta). Filled and open circles represent adsorption and desorption data, respectively (upper). Plot of the linear region of the N_2 isotherm of QUST-82 for the BET and Langmuir equations (bottom).

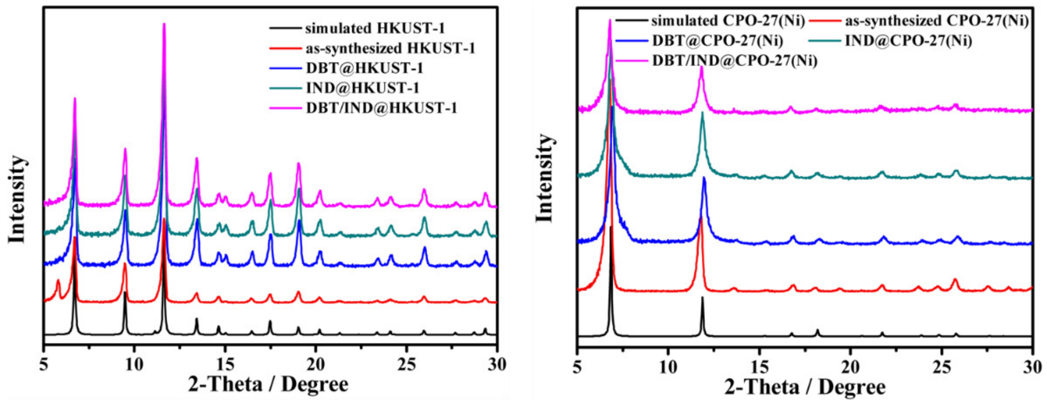

Figure S6. PXRD patterns of HKUST-1 and CPO-27 (Ni) after various treatments.

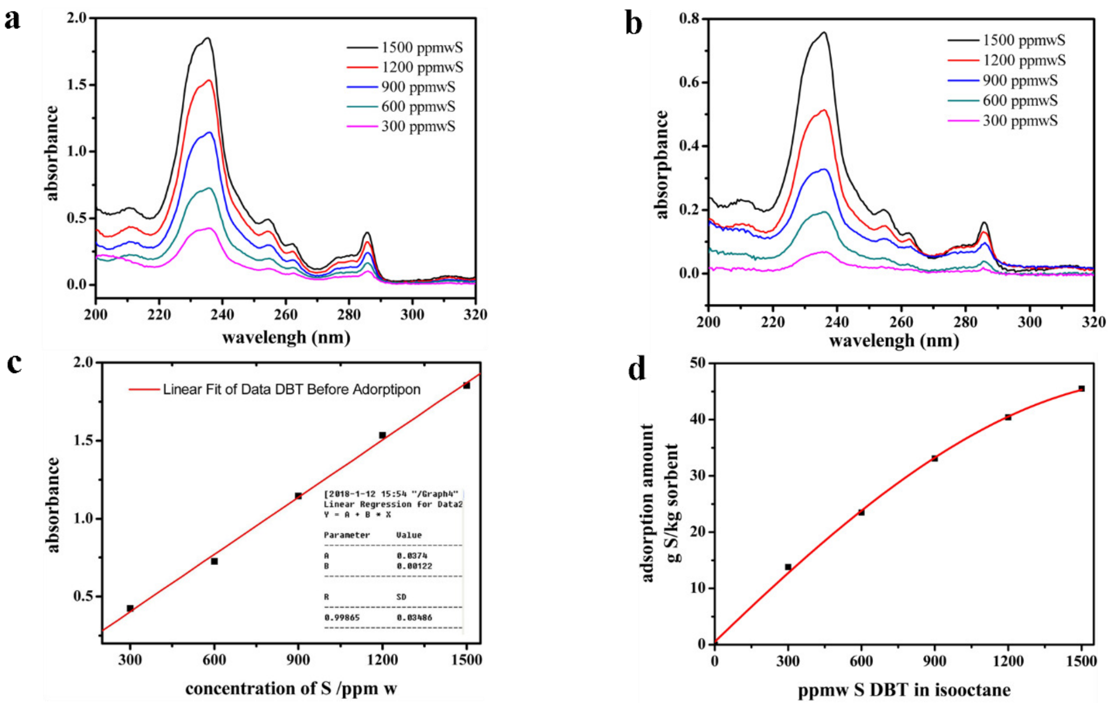

Figure S7. UV spectra of isooctane solution of DBT at different concentrations before (a) and after (b) adsorption; linear fit of standard curve before adsorption (c); adsorption isotherm for DBT from the isooctane solution (d).

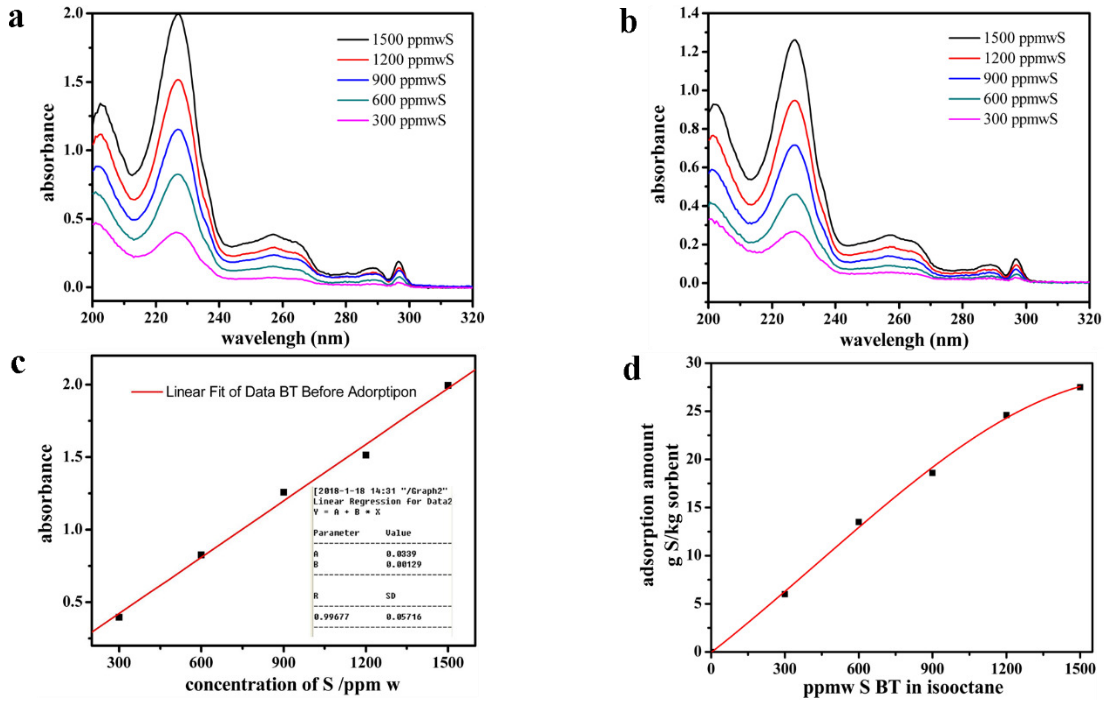

Figure S8. UV spectra of isooctane solution of BT in different concentrations before (a) and after (b) adsorption; linear fit of standard curve before adsorption (c); adsorption isotherm for BT from the isooctane solution (d).

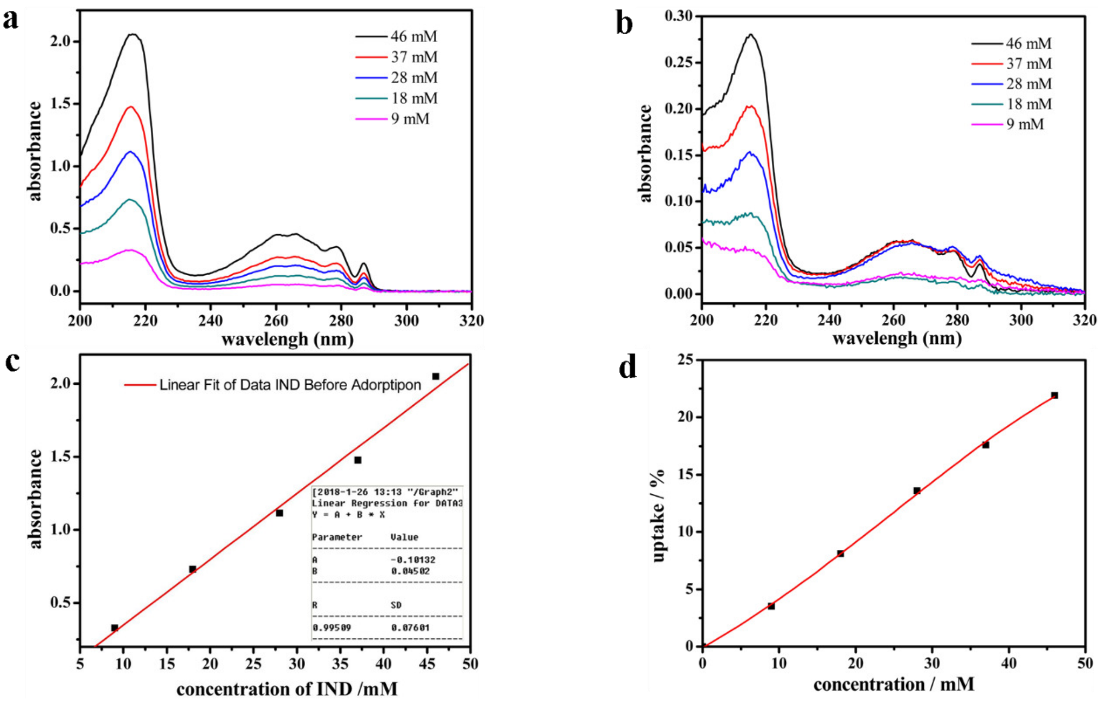

Figure S9. UV spectra of isooctane solution of IND in different concentrations before (a) and after (b) adsorption; linear fit of standard curve before adsorption (c); adsorption isotherm for IND from the isooctane solution (d).

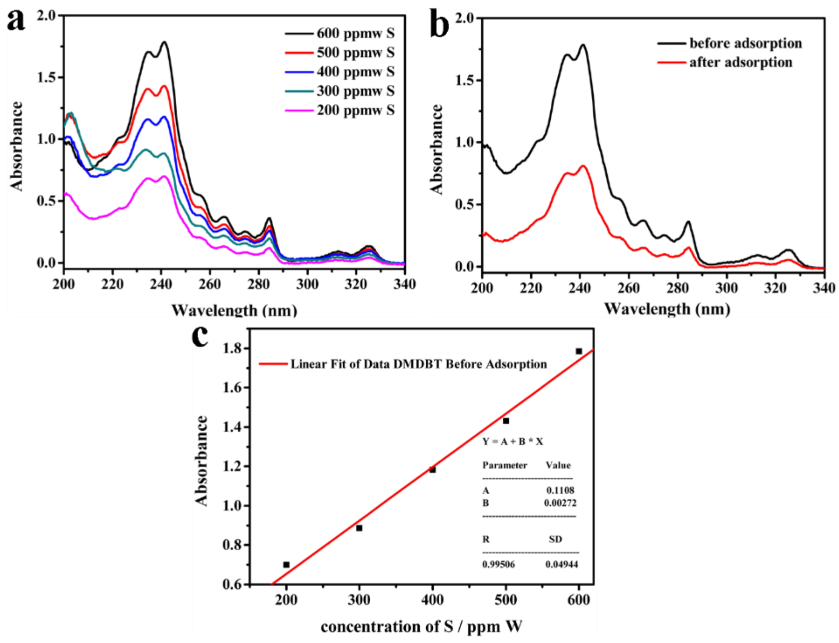

Figure S10. UV spectra of isooctane solution of DMDBT in different concentrations (a);before and after adsorption at 600 ppmw S (b); linear fit of standard curve before adsorption (c).

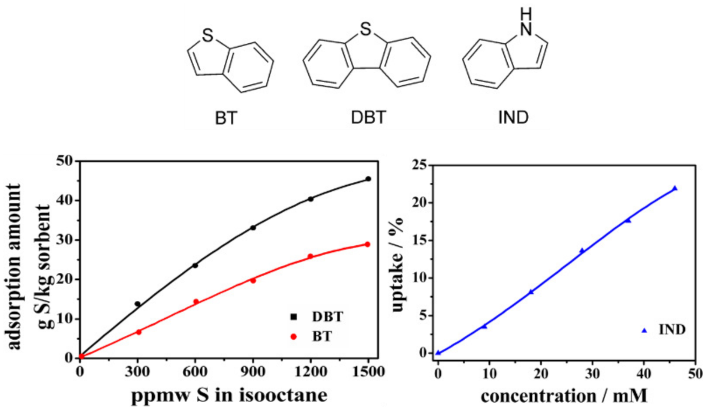

Figure S11. Adsorption isotherms for dibenzothiophene (DBT), benzothiophene (BT) and indole (IND) of QUST-82 from isooctane solution.

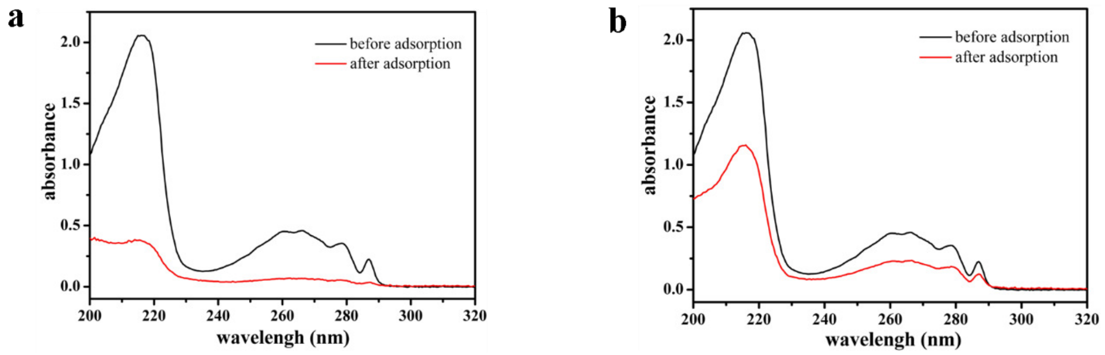

Figure S12. UV spectra of isooctane solution to check the adsorption capacity of HKUST-1 (a) and CPO-27 (Ni) (b) for IND at a concentration of 46 mM.

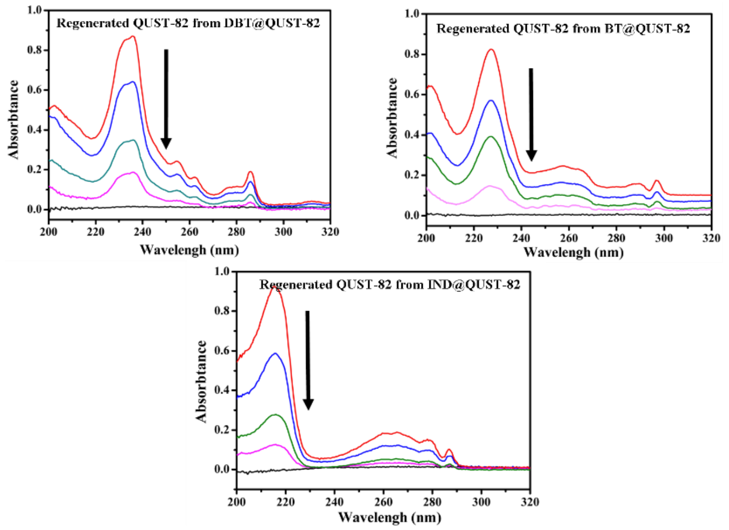

Figure S13. UV spectra for DBT@QUST-82, BT@QUST-82 and IND@QUST-82 during the regeneration experiments (the spectra corresponding to the washed ethanol from top to bottom).

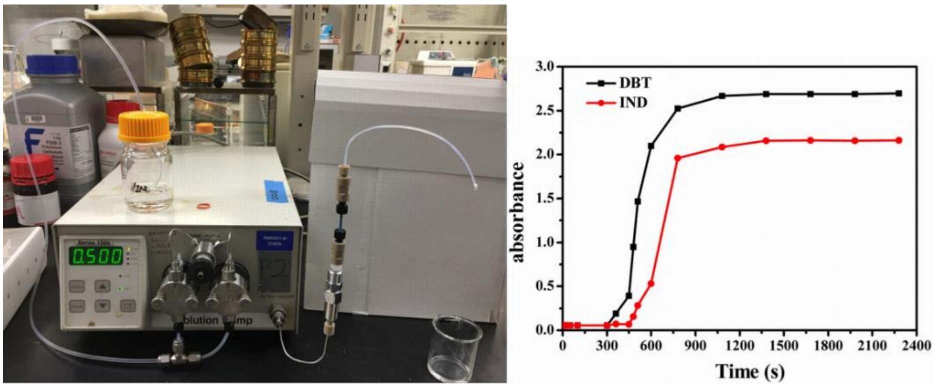

Figure S14. A breakthrough experiment on a QUST-82 column with a mixed solution of DBT and IND at equivalent concentration with DBT/IND = 1500 ppmw S/46 mM.

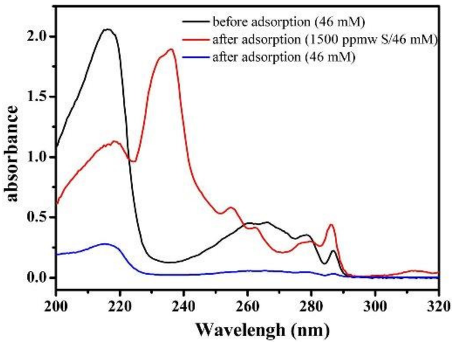

Figure S15. UV spectra of isooctane solution to check the adsorption capacity for IND from a DBT/IND mixture at concentration of 1500 ppmw S/46 mM.

V. Supporting tables

Table S1. Crystal Data and Structure Refinement Details of QUST-81 and QUST-82

$^{a}R_1 = ||F_o| - |F_c|| / |F_o|$. $wR_2 = [w(|F_o^2| - |F_c^2|)^2 / w|F_o^2|^2]^{1/2}$

Identification code	QUST-81	QUST-82
Empirical formula	$\{[Yb_6Cu_{12}(OH)_4(PyC)_{12}(H_2O)_{36}]\cdot(NO_3^-)_{14}\cdot 2.5H_2O\}_n$	$\{[Yb_4O(H_2O)_4Cu_8(OH)_{8/3}(PyC)_8(HCOO)_4]\cdot(NO_3)_{10/3}\cdot 4H_2O\}_n$
Formula weight	4755.92	2673.34
Temperature (K)	298(2)	120(2)
Wavelength (Å)	0.7288	1.54178
Crystal system	cubic	cubic
space group	Fm-3m	Pm-3m
a (Å)	42.724(3)	23.7296(3)
V (Å^3)	77986(16)	13362.0(3)
Z	8	3
D_c (mg·m^{-3})	0.810	0.997
μ/(mm^{-1})	2.644	5.043
F (000)	18380	3802
Theta range for data collection	1.470 to 19.508°	3.73 to 74.28°
Index ranges	-36<=h<=36, -36<=k<=36, -36<=l<=36	-27<=h<=20, -20<=k<=28, -27<=l<=20
Reflections collected	89289	14256
Independent reflections	1354 [R(int) = 0.1313]	2638 [R(int) = 0.0503]
Completeness	99.3%	97.5%
Max. and min. transmission	0.902 and 0.761	1.00000 and 0.66620
Goodness-of-fit on F^2	2.192	0.884
$R_1{}^a$ (I>2sigmaI)	R1 = 0.1325, wR2 = 0.4176	R1 = 0.0737, wR2 = 0.2126
R indices (all data)	R1 = 0.1381, wR2 = 0.4347	R1 = 0.1043, wR2 = 0.2333

Table S2. The adsorption capacities of QUST-82 for DBT, BT and IND at different concentrations.

CONC	DBT		BT	
	g S kg^{-1} MOF	g DBT kg^{-1} MOF	g S kg^{-1} MOF	g BT kg^{-1} MOF
1500 ppmw S	45.5 (43.6)[a]	262 (251)[a]	27.5 (26.3)[a]	116 (111)[a]
1200 ppmw S	40.4	233	24.6	103
900 ppmw S	33.1	192	18.6	78
600 ppmw S	23.5	135	13.5	57
300 ppmw S	13.8	80	6.0	25

CONC	g IND kg^{-1} MOF	wt%
46 mM	219.4 (204.8)[a]	22 (20)[a]
37 mM	175.5	18
28 mM	135.5	14
18 mM	80.7	8.1
9 mM	35.1	3.5

[a] the adsorption amount by using regenerated QUST-82.

Table S3. MOFs Surface Area, Pore Volume, Open Metal Site and adsorption capacities of DBT, BT (1500 ppmw S), DMDBT (600 ppmw S) or IND (46 mM)

MOF	Langmuir Surface Area (m^2/g)	Pore Volume (cm^3/g)	Open Metal Site (OMS)	DBT g kg^{-1} MOF	BT g kg^{-1} MOF	DMDBT g kg^{-1} MOF	IND g kg^{-1} MOF
QUST-82	2733	0.964	Yes	**262**	**116**	**115**	**219**
UMCM-150[a]	3100	1.11	Yes	478	169	272	—
MOF-505[b]	1830	0.63	Yes	225	215	179	—
HKUST-1[c]	2175	0.75	Yes	259	105	106	206
MOF-5[d]	2900	0.59	No	161	51	—	—
MOF-177[e]	4500	1.59	No	92	37	—	—
Cu_3(NAPANA)[f]	961	0.404	Yes	187	117	—	—
CPO-27 (Ni)[g]	1083	0.41	Yes	158	—	—	106

(a) A. G. Wong-Foy, O. Lebel, A. J. Matzger, J. Am. Chem. Soc. **2007**, 129, 15740.

(b) B. Chen, N. W. Ockwig, A. R. Millward, D. S. Contreras, O. M. Yaghi, Angew. Chem. Int. Ed. **2005**, 44, 4745.

(c) A. G. Wong-Foy, A. J. Matzger, O. M. Yaghi, J. Am. Chem. Soc. **2006**, 128, 3494.

(d) H. Li, M. Eddaoudi, M. O'Keeffe, O. M. Yaghi, Nature, **1999**, 402, 276.

(e) H. K. Chae, D. Y. Siberio-Perez, J. Kim, Y. B. Go, M. Eddaoudi, A. J. Matzger, M. O'Keeffe, O. M. Yaghi, Nature, **2004**, 427, 523.

(f) S. L. Li, Y. Q. Lan, H. Sakurai, Q. Xu, Chem. Eur. J. **2012**, 18, 16302.

(g) P. Dietzel, B. Panella, M. Hirscher, R. Blom, H. Fjellvag, Chem. Comm., **2006**, 959.

Table S4. BET Surface area, pore volume and N_2 uptake for QUST-82, regenerated QUST-82, DBT@QUST-82, BT@ QUST-82, IND@QUST-82 and DBT/IND@QUST-82

**MOFs**	**BET Surface area [m^2g^{-1}]**	**Pore Volume [cm^3g^{-1}]**	**N_2 uptake [cm^3g^{-1}]**
QUST-82	2148	0.964	622
Regenerated QUST-82 from DBT@QUST-82	2043	0.914	590
Regenerated QUST-82 from BT@QUST-82	2078	0.928	601
Regenerated QUST-82 from IND@QUST-82	2035	0.886	581
Regenerated QUST-82 from DBT/IND@QUST-82	2032	0.871	576
DBT@QUST-82 (900 ppmw S)	818	0.446	288
BT@QUST-82 (900 ppmw S)	1167	0.569	368
IND@QUST-82 (28 mM)	921	0.505	324
DBT/IND@QUST-82 (1500 ppmw S/46 mM)	483	0.233	154

Table S5. The adsorption capacities for DBT from pure DBT or the mixture of DBT/IND at equivalent concentrations using QUST-82, HKUST-1 and CPO-27 (Ni)

**MOFs**	**CONC**	**DBT**	**g S kg^{-1} MOF**	**g DBT kg^{-1} MOF**	**DBT/IND**	**g S kg^{-1} MOF**	**g DBT[a] kg^{-1} MOF**	**DBT[a]/ DBT %**
**QUST-82**	DBT (ppmw S) IND (mM)	1500	45.5	262	1500/46	41.9	241	92
		300	13.8	79.6	300/9	12.0	69.5	87
**HKUST-1**	DBT (ppmw S) IND (mM)	1500	44.3	255	1500/46	34.1	196	77
		300	13.3	76.6	300/9	6.04	34.8	45
**CPO-27 (Ni)**	DBT (ppmw S) IND (mM)	1500	27.5	158	1500/46	13.9	80.0	51
		300	12.1	69.7	300/9	2.13	12.3	18

[a] the adsorption amount of DBT from the mixture of DBT/IND.
